# Using Delaunay triangulation and Voronoi tessellation to predict the toxicities of binary mixtures containing hormetic compound

**DOI:** 10.1038/srep43473

**Published:** 2017-03-13

**Authors:** Rui Qu, Shu-Shen Liu, Qiao-Feng Zheng, Tong Li

**Affiliations:** 1 Key Laboratory of Yangtze River Water Environment, Ministry of Education, College of Environmental Science and Engineering, Tongji University, Shanghai 200092, China; 2 State Key Laboratory of Pollution Control and Resource Reuse, College of Environmental Science and Engineering, Tongji University, Shanghai 200092, China

## Abstract

Concentration addition (CA) was proposed as a reasonable default approach for the ecological risk assessment of chemical mixtures. However, CA cannot predict the toxicity of mixture at some effect zones if not all components have definite effective concentrations at the given effect, such as some compounds induce hormesis. In this paper, we developed a new method for the toxicity prediction of various types of binary mixtures, an interpolation method based on the Delaunay triangulation (DT) and Voronoi tessellation (VT) as well as the training set of direct equipartition ray design (EquRay) mixtures, simply IDV_equ_. At first, the EquRay was employed to design the basic concentration compositions of five binary mixture rays. The toxic effects of single components and mixture rays at different times and various concentrations were determined by the time-dependent microplate toxicity analysis. Secondly, the concentration-toxicity data of the pure components and various mixture rays were acted as a training set. The DT triangles and VT polygons were constructed by various vertices of concentrations in the training set. The toxicities of unknown mixtures were predicted by the linear interpolation and natural neighbor interpolation of vertices. The IDV_equ_ successfully predicted the toxicities of various types of binary mixtures.

Delaunay triangulation (DT) is a technique for creating a mesh of contiguous, nonoverlapping triangles from a dataset of points^1^. The DT is the geometric dual of the voronoi tessellation (VT)^2^. A Voronoi diagram splits up a plane based on a set of original points. Each polygon, contains an original point and all areas that are closer to that point than any others^3^. VT and DT have been applied in many areas of mathematics and the natural sciences^4^,^5^,^6^. For example, people used linear interpolation (based on DT) and natural neighbor interpolation (based on VT) to predict the climate data or geographic data^7^,^8^. Natural neighbor interpolation and linear interpolation are the methods of spatial interpolation. Since data acquired in practical production are always limited, spatial interpolation is an effective way to remedy data. Mixture toxicity researching are also faced with the same problem, it is impossible to test the toxicity of all mixtures. This is because multicomponent mixture is a very complex system, the concentration and proportion of components are infinite^9^.

People often use concentration addition (CA) and independent action (IA) to predict the toxicity of the mixture^10^,^11^,^12^. In particular, CA was proposed as a reasonable default approach for ecological risk assessment of chemical mixtures^13^. CA can be expressed as shown in equation (1):


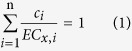


where *n* refers to the number of components of mixture, *c*_*i*_ denotes the concentration of the *i*th component in the mixture that elicits *x*% effect, and *EC*_*x,i*_ is the effective concentration of the component *i* that provokes the same effect (*x*%) when applied alone. The fraction *c*_*i*_/*EC*_*x,i*_ is often termed a ‘toxic unit (*TU*_*i*_)’, and CA is hence also known as ‘toxic unit summation’^14^.

CA is based on the premise that all components have similar mechanisms of action (MOA)^15^. IA assumes that components of mixture act dissimilarly^16^. However, toxicologists often know little about the MOAs of substances^17^. There is an obvious defect of CA, the only condition for CA predicting the effective concentrations (EC_*x*_) is that all components have definite effective concentrations at the given effect (*x*%). Therefore, CA cannot predict the toxicity of mixture at some effect zones (predictive blind zones) when the mixture and the components have different effects^18^, such as some compounds induce hormesis^19^. Hormesis is a concentration-response phenomenon that is characterized by low-dose stimulation and high-dose inhibition^20^. IA can predict mixture effect when all or some of the chemicals induce hormesis in theory, but in practice, some people think IA will lose its conceptual framework if negative values for single chemical effect were involved^21^. The calculation for the IA model can be performed according to equation (2):


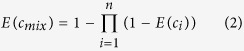


where *E(c*_*mix*_) is the predictive effect of a mixture with a total concentration of *c*_*mix*_, *c*_*i*_ is the individual concentration of *i*th compound in the mixture and *E(c*_*i*_) is the effect of this concentration if the compound is applied singly. In fact, it isn’t uncommon for the toxicity of a mixture deviates from such predictions when antagonism or synergism occurs. If the toxicological interaction, antagonism or synergism, is identified by CA at a certain mixture ratio or concentration level, CA can not certainly predict the toxicity accurately at other mixture ratio or concentration level. Because the interaction is mixture ratio-dependent and concentration level-dependent^22^,^23^. For example, we have determined the mixture toxicities of 1-ethyl-3-methylimidazolium chloride ([emim]Cl) and metalaxyl (MET) on *Vibrio qinghaiensis* sp.-Q67 (*V. qinghaiensis*), and found that the mixture exhibited synergism at one mixture ratio, but showed antagonism at another ratio^24^. It means that toxicological interaction of chemicals does occur neither uniformly nor predictably. Besides CA and IA, some researchers tried to predict the toxicity of mixture through using the QSAR-based approaches to predict the toxicities of single compound and then compute the mixture descriptors^25^,^26^,^27^. However, QSAR is very successful in dealing with individual compounds, but how to calculate rationally the mixture descriptors is still in dispute.

In this paper, we developed a new method for the toxicity prediction of various types of binary mixtures (Fig. 1), an interpolation method based on the DT and VT as well as the training set of direct equipartition ray design (EquRay) mixtures, simply IDV_equ_. In order to obtain the required data, time-dependent toxicity of two ILs, three pesticides and binary pesticide-IL mixtures on *V. qinghaiensis* were determined. Because we aim to test the predictive capability of the methods when toxicological interaction occurs, some results of our previous researches were used. They are the mixture toxicities of [emim]Cl and MET, and mixture toxicities of 1-ethyl-3-methylimidazolium bromine ([emim]Br) and MET^24^.

## Results

### Concentration-response curves (CRCs) of five single substances

The concentration-response curves (CRCs) of three pesticides and two ILs at seven exposure times were displayed in Fig. 2. For three pesticides, CRCs are S-shaped during the exposure times. [epy]Cl and [epy]Br show hormetic effects from 4 to 12 h. The *E*_*min*_s (minimal effect) of [epy]Cl and [epy]Br increase from 4 to 12 h. Concentration-response models, statistics (determination coefficient, R^2^, and root mean square error, RMSE), EC_30,_ EC_50,_ EC_70_ and characteristic parameters (zero effect point (*ZEP*), minimum effective concentration (*EC*_*min*_) and *E*_*min*_ for J-shape CRCs)^28^,^29^ of pesticides and ILs at seven exposure times were reported in the Table S1. The experimental concentration-inhibition points, fitted CRCs of five compounds and 95% confidence intervals (CI) at seven exposure times were shown in Figure S1.

### Toxicities of 30 mixture rays

17 of 30 mixture rays in six binary systems, [epy]Br-DOD, [epy]Br-MET, [epy]Br-SIM, [epy]Cl-DOD, [epy]Cl-MET and [epy]Cl-SIM, show hormesis at different times. They are the R1, R2, R3 and R4 of [epy]Br-DOD systems; R1, R2 and R3 of [epy]Br-MET; R1 and R2 of [epy]Br-SIM; R1, R2 and R3 of [epy]Cl-DOD systems; R1, R2 and R3 of [epy]Cl-MET; R1 and R2 of [epy]Cl-SIM. The CRCs of 30 mixture rays were shown in Figure S2.

The toxicities of 20 mixture rays in the four systems, [epy]Br-MET, [epy]Br-SIM, [epy]Cl-MET and [epy]Cl-SIM, are additive at seven times and various effect levels because all the effect residual ratios (ERRs) are almost zero. For the ten mixture rays in the other two system, [epy]Br-DOD and [epy]Cl-DOD, some mixtures (mainly at higher effect levels and shorter exposure times) have no toxicological interactions, the other ones (mainly at lower effect levels and longer exposure times) produce significant antagonism (seeing the ERR values in Table 1. For example, the R3 ray in [epy]Br-DOD systems, has no interaction at the exposure times of shorter than 4 h and the effect levels of larger than 60%, the corresponding ERR values basically close to zero and exihibit antagonism at the times of longer than 4 h and the effects of less than 60%, the corresponding ERRs significantly less than zero.

### Performance of IDV_equ_

The accuracy rate (AR), R^2^ and RMSE of IDV_equ_ predicting the toxicities of various mixtures in eight binary mixture systems by the leave-many-out cross-validation (LMOCV) and leave-one-out cross-validation (LOOCV) analysis were displayed in Table 2. For LMOCV analysis, AR of linear interpolation (LinIP) are all more than 90% except for [emim]Cl-MET. The maximal AR of LinIP is 96.71% for [epy]Br-DOD. The minimal AR of LinIP is 89.93% for [emim]Cl-MET. The R^2^ ranges from 0.9665 to 0.9819. The AR values of natural neighbor interpolation (NeiIP) are all more than 90% except for [epy]Cl-MET and [emim]Cl-MET. The maximal AR of NeiIP is 97.00% for [epy]Br-DOD. The minimal AR of NeiIP is 88.71%, The R^2^ ranges from 0.9623 to 0.9812.

For LOOCV analysis, the ARs of LinIP has high variance and ranges from 73.81% to 95.00%. The maximal AR occurs in [epy]Br-DOD. The minimal AR occurs in [epy]Br-SIM. The ARs of NeiIP also has high variance and ranges from 73.10% to 94.76%. Like LinIP, the maximal AR of NeiIP occurs in in [epy]Br-DOD. The minimal AR occurs in [epy]Br-SIM.

In Table 2, the toxicities of all mixture rays in the former four systems, [epy]Br-MET, [epy]Br-SIM, [epy]Cl-MET and [epy]Cl-SIM, are additive at seven exposure times and various effect levels. The plots of the predictive toxicities by the LMOCV based on LinIP and NeiIP vs. observation toxicities were showed in Figure S3a and S3b. Similarly, the predictive plots by the LOOCV were showed in Figure S4a and S4b. It was shown that some mixtures in [emim]Br-MET and [emim]Cl-MET^24^ show synergism while some mixtures in [epy]Br-DOD and [epy]Cl-DOD produce antagonism, which implies the toxicities of the mixtures cannot be predicted by CA. However, IDV_equ_ can predict the toxicities of the mixtures not only having no toxicological interaction (the former four systems) but also having synergism or antagonism (the latter four systems). The plots of the predictive toxicities by the LMOCV based on LinIP and NeiIP vs. observation toxicities for [emim]Br-MET, [emim]Cl-MET, [epy]Br-DOD and [epy]Cl-DOD at seven exposure times were showed in Figure S5a and S5b. Similarly, the predictive plots by LOOCV were showed in Figure S6a and S6b. The predictive ability of LinIP and NeiIP is almost the same. However, the results from LMOCV and LOOCV are different. The ARs from LOOCV analysis are, as a whole, lower than those from LMOCV.

## Discussion

### IDV_equ_ is independent of the chemical MOA

When CA or IA is used to predict the toxicity of the mixture, people need to know the MOA of various mixture components. However, the MOA of most chemicals still remain unknown, it will result in difficulties to choose CA or IA as the reference models^30^. Furthermore, real-world mixtures are made up of chemicals with both similar and dissimilar MOA^31^. Although the IDV_equ_ requires some data of mixture toxicity, it doesn’t require the MOA of each chemical. IDV_equ_ is based on the experiment datas, it is more reliable because no model can guarantee the correctness of the prediction.

### IDV_equ_ can predict the toxicity of various types of binary mixtures

[epy]Br, [epy]Cl and some mixture rays show hormesis at different exposure times in this study. Predicting the hormesis induced by mixture has been a hot topics in toxicological sciences, because hormesis is a very common phenomenon^32^,^33^ and contaminants always co-occour in ecosystems^16^. The results indicated that IDV_equ_ can predict the toxicity of binary mixtures which contain hormetic chemical well. The results of this paper also show that, even if interaction occurs, IDV_equ_ can also well predict the toxicity at other ratios or concentrations. But if interaction is identified by CA at a certain ratio or concentration, whether CA can predict the toxicity accurately at other ratios or concentrations is unknown.

Our previous research^24^ and the results of this paper showed that toxicological interaction in the pesticide-IL mixture system is ratio-dependent and time-dependent. An empirical model was formulated by Jonker *et al*.^34^ to determine the magnitude of interaction, it requires experimental designing which covers all ratios and concentration for optimal performance. Unforunately, the combination ratio and concentration of mixtures is infinite^16^,^35^,^36^. In this paper, IDV_equ_ predict the interaction just need the toxicity of five mixture rays and two single compounds. It will minimising the experimental investigations and the consequent consumption of time and resources.

### Largest interactions does not always occur at equitoxic ratios

Some people think the largest interactions most often occur at equitoxic ratios. But the results of this paper are not consistent with this hypothesis. In [epy]Br-DOD system, the maximum ERR occur at R3 (0.25 h), R2 (2 h), R3 (4 h), R3 (6 h), R4 (8 h), R4 (10 h) and R4 (12 h). In [epy]Cl-DOD system, the maximum ERR occur at R3 (0.25 h), R3 (2 h), R4 (4 h), R4 (6 h), R3 (8 h), R4 (10 h) and R4 (12 h). According to EquRay, the mixture ratio of R3 is 1:1 base on EC_50_ (equitoxic ratio), but the maximum ERR don’t always occur at R3. There are a lot of researches showed that the largest interactions don’t often occur at equitoxic ratios. For example, people tested the binary mixture of zinc and copper on *Tympanotonus fuscatus* and found that the mixture (1:4 mixture ratio base EC_50_) exhibited strong antagonism, but the mixture showed additive at equitoxic ratio^37^. Cedergreen^38^ invertigated the binary mixture of mecoprop and terbuthylazine on the floating plant *Lemna minor* and found that antagonism was larger for mixtures with higher proportions of mecoprop.

### There are no predictive blind zones for IDV_equ_

CA cannot predict the toxicity of mixture at some effect zones (predictive blind zones) when the compounds share a different range of effects. The predictive blind zones contains a variety of types: partial mixture components can cause hormesis; all mixture components can horemsis but their E_min_ is not equal; the maximum effect of a mixture is different from that of a single component. In these cases, IDV_equ_ is able to predict the toxicity of the mixture at all effect zones. Although a generalized concentration addition (GCA) model can predict the toxicity of mixtures when some chemicals have a smaller maximum effect level than others^39^, it need assume that all CRCs were fitted by Hill functions and slope parameter was one. This limits the use of the GCA model.

People always hope that a model can be applied to all situations. For example, some people proposed a modified model and automated fitting procedure to describe CRCs with multiphasic features^40^. The application of IDV_equ_ is not restricted by the CRC of single compounds and mixtures. There are no predictive blind zones for IDV_equ_, it should be a universally applicable method for predicting the mixture toxicity. Theoretically, LinIP and NeiIP method can predict the toxicity of multi-component mixtures. In fact, we have studied the toxicity of multi-component mixtures in our previous works. For example, we used UD-Ray method to select the representative mixtures from a lot of mixtures rays in the multi-component mixture system^41^. But the interpolation methods to predict the toxicity of multi-component mixtures need to be further validated. We expect IDV_equ_ to be a tool useful for experimentalists and analysts interested in the study of mixture toxicity, because people are rarely exposed to a single hazardous chemical^42^.

## Materials and Methods

### Test compounds

The test chemicals are: 1-ethylpyridinium Chloride ([epy]Cl, highly soluble in water), 1-ethylpyridinium Bromide ([epy]Br, highly soluble in water), dodine (DOD, H_2_O solubility: 63 mg/L at 25 °C), metalaxyl (MET, H_2_O solubility: 8400 mg/L at 22 °C), simetryn (SIM, H_2_O solubility: 450 mg/L at 22 °C). Two ILs were purchased from TCI (Japan) and three pesticides were purchased from Dr.ehrenstorfer (Germany). All solutions were prepared with Milli-Q water and stored in darkness at 4 °C before test. Some physiochemical properties, CAS number, concentration of stock, H_2_O solubility and source of five chemicals (Table S2).

### Mixture design

Like our previous researches^24^, each binary mixture system contains five mixture rays (noted as R1, R2, R3, R4 and R5). Six binary mixtures were: [epy]Br-DOD, [epy]Br-MET, [epy]Br-SIM, [epy]Cl-DOD, [epy]Cl-MET and [epy]Cl-SIM. EquRay was employed to design the basic concentration compositions of five binary mixture rays^43^. The mixture ratios (*p*_*i,j*_)^44^, the ratio of the concentration of the *j*th component in the *i*th ray to the total concentration of the ray, of various components in 30 mixture rays and the concentrations of stocks were listed in Table 3.

### Determine the toxicities of single components and mixture rays

**Figure 1:**
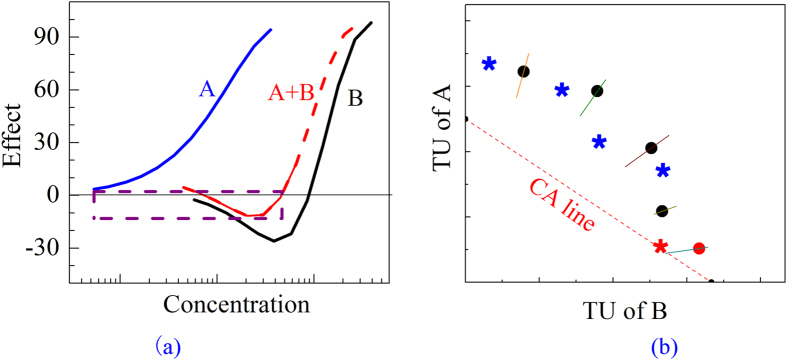
The area where CA cannot predict ((**a**) in the dash box; (**b**) not on the CA line) where TU refers to toxic unit.

**Figure 2:**
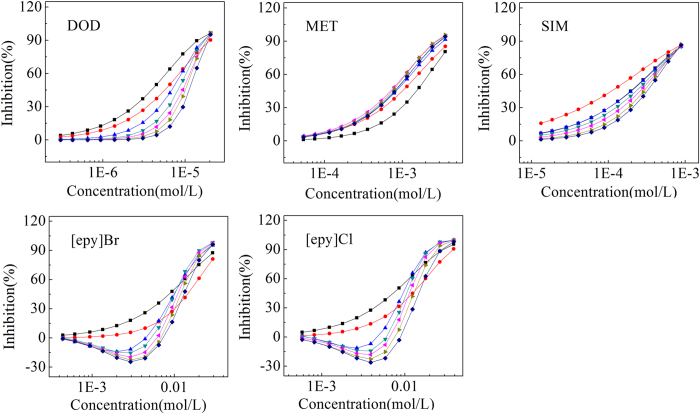
The concentration-inhibition curves of three pesticides and two ionic liquids at seven exposure times (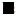: 0.25 h; 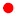: 2 h; 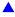: 4 h; 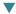: 6 h; 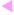: 8 h; 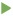: 10 h; 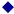: 12 h).

A time-dependent microplate toxicity analysis was given in our previous works^45^. The toxic effects of single components and mixture rays at different times and various concentrations were determined by it. The setup of controls and treat-group in the 96-well microplate is designed according to Fig. 1 in the literature^46^. The 12 concentration gradients were calculated according to equation 3 in in our previous work^47^. The freeze-dried *V. qinghaiensis* was purchased from Beijing Hamamatsu Corp., Ltd. (Beijing, China). The relative light unit (RLU) of every well was determined on the Power-Ware microplate spectrophotometer (American BIO-TEK Company) at 22 ± 1 °C. During exposure, readings were taken at 0.25, 2, 4, 6, 8, 10 and 12 h. Inhibition ratio of bioluminescence was used to characterize the toxicity, noted as E:


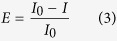


where *I*_*0*_ is the average RLU of *V. qinghaiensis* exposed to the experimental group and *I* indicates the average RLU of *V. qinghaiensis* exposed to controls.

### Construct DT triangles and VT polygons

**Figure 3:**
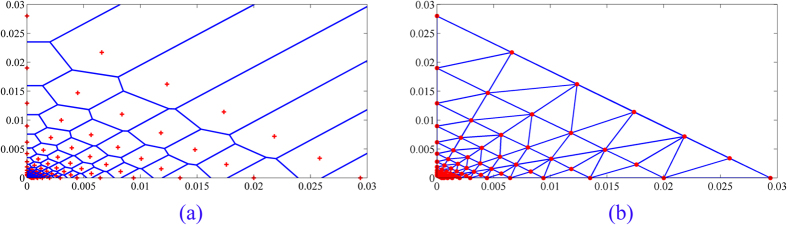
The voronoi diagrams (**a**) and delaunay triangulation (**b**) of 84 test points (two single compounds and five mixture rays).

The concentration-toxicity data of the pure components and various mixture rays were acted as a training set. The DT triangles^7^ and VT polygons^48^ were constructed by various vertices of concentrations in the training set. Each single compound or mixture ray contain 12 different test concentrations, so the two single compounds and five mixture rays contain 84 test points. VT is the geometric dual structure of the DT^49^. The VT and DT of 84 vertices were displayed in Fig. 3.

### Using LinIP and NeiIP to predict the toxicity of unknown mixtures

**Figure 4:**
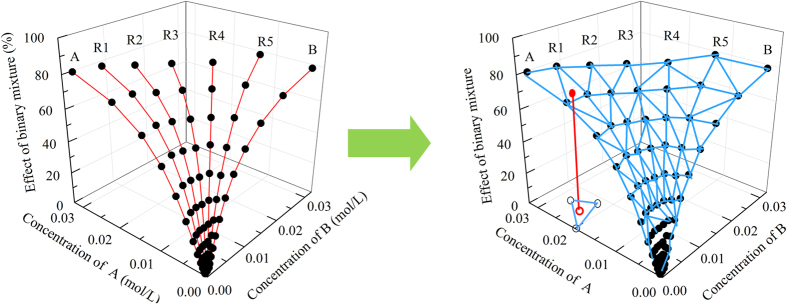
Sketch-map of Delaunay triangulation for predicting the toxicity of binary mixture. (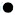: experimental values; 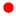: query point; R1~R5: five mixtures rays with different ratio; A and B: two components of the binary mixture).

The toxicities of unknown mixtures were predicted by the LinIP and NeiIP of vertices. The sketch-map of LinIP and natural NeiIP for predicting the toxicity of binary mixture is displayed in Fig. 4.

Firstly of all, define the point P like this: (X_i_,Y_i_,Z_i_), X_i_ means concentration of one substance, Y_i_ means concentration of another chemical, Z_i_ means the toxicity of the binary mixture. LinIP is used to interpolate the toxicity of query point. The operation steps of LinIP are as follows^7^: each triangle covers an area and the toxicity of query points in this area is predicted based on the triangle that covers it. Let P_1_, P_2_, and P_3_ be the three vertices of the triangle, located at P_1_ = (X_1_,Y_1_,Z_1_), P_2_ = (X_2_,Y_2_,Z_2_) and P_3_ = (X_3_,Y_3_,Z_3_). Suppose that the plane which is defined by the three points is given by


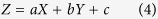


We will obtain a linear system of equations by inserting the X, Y and Z values of each of the three known points into this equation^50^:


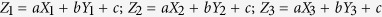


where the coefficients a, b and c of the equation (4) can be found when we solve this system of equations. The value of Z for any query point within this triangle will be available after the above steps.

Although voronoi tesselation of given scattered point set has been constructed first, when a query point is inserted a new voronoi polygon is created which overlaps the original voronoi^48^. The intersection of the new Voronoi polygon with the original Voronoi diagram is defined^51^ as follows:


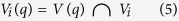


where *V*_*i*_ is the Voronoi polygon of the *i*th natural neighbor *x*_*i*_. It is said that the new Voronoi polygon *V(q)* overlaps some area from the neighboring Voronoi tiles *V*_*i*_ of the query point. Then, the weight of *i*th natural neighbor, W*i*, is defined by


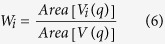


where *Area* [*V*_*i*_*(q)*] is the area of each intersection Vi(q). The *Area* [*V(q)*] is the total area of the polygon *V(q)*. Then, the NeiIP of the query point q and is defined by


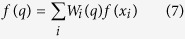


where *f(x*_*i*_) is the function value in the *i*th natural neighbor point. *f(q)* is the function value of the query point.

### Validation of the predictive capabilities of IDV_equ_

LMOCV and LOOCV were selected to determine the performance of IDV_equ_. LMOCV randomly splits the dataset into training and validation data, approximately 2/3 of the data was allocated to the training set while the remaining 1/3 was allocated to the test set^52^. In this paper, mixture toxicity (n = 60) were randomly divided into two disjoint parts; 1/3 of the combined toxicity (n = 20) was allocated to the test set, 2/3 of the combined toxicity (n = 40) and single toxicity (n = 24) were combined resulting in a training set. The holdout method was repeated 20 times with randomly selected training and holdout sets^53^.

LOOCV is a particular case of LMOCV where M = O (one). In this paper, the toxicity of a mixture ray from the mixture system is selected as a validation set. The toxicity of remaining four mixture rays and two individual substances are used as the training set. The purpose of this is to test whether the two methods can accurately predict the complete CRCs.

The predicted values were compared with 95% CI of observed value. If CI overlaps predictions, we believe that the predicted value is correct.The root mean square error (RMSE), coefficient of determination (R^2^) and accuracy rate (AR) were used to assess the performance. Accuracy rate (AR) is the number of query point be predicted correctly divided by the number of total query point.

### CRCs fitting and toxicity interaction characterization

Monotonic S-shaped CRCs were fitted by Logit or Weibull function^54^. A five parameters logistic equation (equation 8) was chosen to describe the non-monotonic J-shaped CRCs^55^. CA was used to predicted the toxicity of mixtures rays.


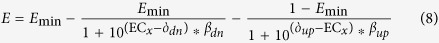


where *E*_*min*_ refers to the minimum effect or maximum stimulatory effect, *ε*_*dn*_ refers to the concentration at the effect of *E*_*min*_/2 in the falling section (negative slope), *β*_*dn*_ refers to the slope at the point (*ε*_*dn*_, *E*_*min*_/2), *ε*_*up*_ refers to the median effective concentration, *β*_*up*_ to the slope at the point (*ε*_*up*_, 50), EC_x_ refers to concentration.

*ERR*_*x*_ (equation 9) was used to quantify the toxicological interaction (synergism or antagonism) in mixtures^24^. Considering the CI, the value of *ERR*_*x*_ at a specific effect (*x*) can be computed as follows:


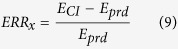


where *E*_*CI*_ is the effect (toxic response) corresponding to the upper limit (when antagonism occurs) or lower limit (when synergism occurs) of CI, *E*_*prd*_ is the effect value predicted by an additive reference model such as concentration addition (CA) at the same concentration (*EC*_*x*_). When *ERR*_*x*_>, =, and <0, say the mixture at the effect of *x* being synerigism, additive action, and antagonism.

## Additional Information

**How to cite this article:** Qu, R. *et al*. Using Delaunay triangulation and Voronoi tessellation to predict the toxicities of binary mixtures containing hormetic compound. *Sci. Rep.* **7**, 43473; doi: 10.1038/srep43473 (2017).

**Publisher's note:** Springer Nature remains neutral with regard to jurisdictional claims in published maps and institutional affiliations.

## Supplementary Material

Supplementary Information

## Figures and Tables

**Table 1:** Effect residual ratios (%) of different effect levels of [epy]Br-DOD and [epy]Cl-DOD mixture rays at seven exposure times.

Ray	Effect (%)	[epy]Br-DOD							[epy]Cl-DOD						
		0.25 h	2 h	4 h	6 h	8 h	10 h	12 h	0.25 h	2 h	4 h	6 h	8 h	10 h	12 h
R1	10	—	—	—	−0.99	−0.44	—	−33.78	—	−6.03	−29.43	−1.79	−18.35	−33.64	−20.54
	20	—	—	—	—	—	—	−26.15	−2.96	−13.16	−22.08	−1.77	−12.83	−23.42	−14.60
	30	—	—	—	—	—	—	−20.46	−7.12	−12.48	−16.34	−1.42	−8.98	−16.47	−9.71
	40	—	—	—	—	—	—	−15.58	−7.07	−10.02	−10.97	−0.31	−5.79	−10.94	−5.55
	50	—	—	—	—	—	—	−10.80	−5.63	−6.44	−5.80	—	−2.58	−6.24	−2.01
	60	—	—	—	—	—	—	−5.83	−3.70	−2.05	−0.39	—	—	−2.32	—
	70	—	—	—	—	—	—	−0.45	−1.74	—	—	—	—	—	—
	80	—	—	—	—	—	—	—	—	—	—	—	—	—	—
R2	10	−2.86	−3.92	—	−11.14	−27.66	−4.36	−16.46	−7.53	−34.22	−15.46	−41.25	−47.42	−52.88	−45.13
	20	−6.81	−3.16	—	−6.48	−19.79	—	−6.33	−19.70	−32.13	−17.15	−32.76	−37.77	−42.56	−36.15
	30	−6.05	−0.10	—	−3.36	−14.53	—	—	−21.92	−27.85	−15.71	−26.69	−30.77	−34.54	−29.60
	40	−4.51	—	—	−1.00	−10.19	—	—	−21.32	−23.32	−11.47	−21.68	−25.07	−27.93	−24.00
	50	−2.14	—	—	—	−6.66	—	—	−19.05	−18.81	−6.26	−16.97	−19.91	−22.00	−18.54
	60	—	—	—	—	−3.24	—	—	−15.85	−13.27	−1.92	−12.28	−14.91	−16.05	−12.91
	70	—	—	—	—	—	—	—	−12.03	−9.91	−1.24	−7.37	−9.73	−9.82	−6.80
	80	—	—	—	—	—	—	—	−7.928	−4.235	—	−1.85	−3.841	−3.055	−0.35
R3	10	−0.66	—	−36.89	−52.62	−43.78	−56.65	−57.74	−20.09	−50.46	−44.16	−38.06	−59.91	−54.55	−49.19
	20	−15.83	—	−33.80	−45.79	−41.21	−45.98	−47.10	−34.21	−41.81	−41.60	−38.65	−50.90	−47.46	−43.56
	30	−18.00	—	−26.46	−37.29	−32.94	−36.81	−37.73	−35.15	−34.00	−35.84	−34.39	−43.67	−41.14	−37.78
	40	−16.33	—	−19.80	−28.53	−25.46	−28.46	−28.66	−32.22	−26.80	−30.01	−29.88	−37.15	−34.56	−31.61
	50	−13.35	—	−13.09	−20.30	−18.29	−19.94	−19.83	−27.31	−20.81	−24.99	−24.95	−30.74	−27.62	−24.94
	60	−9.35	—	−6.29	−12.26	−11.06	−11.43	−11.08	−21.14	−14.58	−19.32	−18.82	−23.89	−20.10	−17.69
	70	−4.01	—	-	−4.32	−5.87	−2.71	−2.86	−14.48	−10.34	−12.51	−11.68	−16.26	−11.97	−10.52
	80	—	—	—	—	—	—	—	−6.464	−5.30	−6.36	−5.40	−6.04	−1.36	−3.30
R4	10	—	—	−18.04	−41.76	−58.66	−63.89	−63.10	—	−5.55	−57.52	−71.08	−57.74	−64.27	−61.59
	20	—	—	−22.47	−39.92	−49.23	−52.91	−55.40	—	−12.00	−50.65	−61.89	−50.33	−54.33	−51.43
	30	—	—	−19.21	−34.38	−40.40	−44.06	−48.15	−6.06	−10.75	−43.28	−53.03	−43.23	−44.64	−41.90
	40	−2.78	—	−15.05	−27.92	−32.56	−35.84	−40.89	−9.78	−7.28	−35.31	−44.59	−35.43	−35.24	−32.76
	50	−4.06	—	−9.88	−21.39	−24.97	−27.73	−33.64	−9.69	−2.85	−27.38	−36.20	−27.02	−25.95	−23.76
	60	−2.79	—	−4.11	−14.75	−18.11	−19.67	−26.00	−7.13	—	−19.35	−27.64	−18.37	−16.58	−14.77
	70	—	—	—	−8.76	−10.78	−11.44	−16.50	−3.11	—	−12.00	−17.92	−8.48	−6.04	−5.79
	80	—	—	—	−1.53	−2.98	−3.09	−5.59	—	—	−3.00	−7.287	—	—	—
R5	10	—	—	—	−4.28	−42.37	−51.49	−6.68	—	—	−17.70	−40.05	−44.33	−56.09	−46.07
	20	—	—	—	−15.22	−38.11	−43.31	−14.78	—	—	−22.41	−36.47	−42.17	−47.29	−41.91
	30	—	—	—	−16.09	−29.35	−37.14	−18.10	—	—	−19.62	−32.46	−34.90	−40.17	−38.32
	40	—	—	—	−13.85	−22.00	−31.27	−16.58	—	—	−16.05	−26.31	−28.47	−33.23	−31.43
	50	—	—	—	−10.16	−15.22	−24.47	−12.20	—	—	−11.60	−20.21	−22.23	−25.67	−24.41
	60	—	—	—	−5.66	−8.59	−17.37	−6.69	—	—	−6.31	−13.83	−16.44	−17.98	−17.17
	70	—	—	—	−1.33	−4.74	−9.85	−0.06	—	—	−0.27	−8.39	−10.46	−9.99	−9.58
	80	—	—	—	—	—	−1.93	—	—	—	—	−2.57	−3.43	−1.51	−1.51

— refers to the effect predicted by CA is located between the 95% confidence interval (CI) of observed effect.

**Table 2:** Leave-20-out cross validation and leave-one-out analysis to determine the accuracy of linear interpolation and natural neighbor interpolation for predicting the toxicities of binary mixtures.

Mixture system	Leave-20-out						Leave-one-out					
	LinIP			NeiIP			LinIP			NeiIP		
	AR (%)	RMSE	R^2^	AR (%)	RMSE	R^2^	AR (%)	RMSE	R^2^	AR (%)	RMSE	R^2^
[epy]Br-MET	92.14	4.794	0.9811	91.68	4.993	0.9808	87.62	4.739	0.9812	88.33	4.663	0.9826
[epy]Br-SIM	92.14	6.041	0.9665	91.00	6.419	0.9623	73.81	8.050	0.9446	73.10	8.073	0.9457
[epy]Cl-MET	91.96	5.474	0.9797	89.79	5.915	0.9772	90.95	4.891	0.9852	90.48	5.250	0.9836
[epy]Cl-SIM	92.86	6.019	0.9680	92.43	6.126	0.9668	80.95	6.788	0.9669	80.00	7.030	0.9655
[epy]Br-DOD	96.71	4.779	0.9795	97.00	4.892	0.9785	95.00	5.417	0.9725	94.76	5.472	0.9700
[epy]Cl-DOD	93.71	5.381	0.9733	93.39	5.452	0.9726	90.24	6.049	0.9657	90.00	6.047	0.9659
[emim]Br-MET	92.32	5.505	0.9819	90.79	5.709	0.9812	90.48	5.788	0.9803	88.81	6.023	0.9791
[emim]Cl-MET	89.93	5.977	0.9778	88.71	6.081	0.9782	86.67	6.366	0.9738	86.90	6.364	0.9758

RMSE: root mean square error;

R^2^: coefficient of determination;

AR: accuracy rate;

LinIP: linear interpolation;

NeiIP: natural neighbor interpolation.

The toxicity of [emim]Br-MET and [emim]Cl-MET are from our previous research^24^.

**Table 3:** Concentration ratios or mixture ratio (*p*_*i,j*_) of five components (*j* = 1, 2, 3, 4, 5) in 30 mixture rays (i = 1, 2, 3, …, 30) and the concentrations of stocks of various mixture rays.

Ray	Mixture ray	*p*_*i,j*_ (i = 1, 2, …, 30; j = 1, 2, 3, 4, 5) (%)					Concentration of stock (mol/L)
		[epy]Br	[epy]Cl	DOD	MET	SIM	
R1	[epy]Br-DOD-R1	99.98		0.02			4.71E-02
R2	[epy]Br-DOD-R2	99.96		0.04			3.63E-02
R3	[epy]Br-DOD-R3	99.91		0.09			2.62E-02
R4	[epy]Br-DOD-R4	99.83		0.17			1.69E-02
R5	[epy]Br-DOD-R5	99.57		0.43			8.18E-03
R6	[epy]Br-MET-R1	98.68			1.32		5.36E-02
R7	[epy]Br-MET-R2	96.75			3.25		4.76E-02
R8	[epy]Br-MET-R3	93.71			6.29		4.04E-02
R9	[epy]Br-MET-R4	88.17			11.83		3.17E-02
R10	[epy]Br-MET-R5	74.88			25.12		2.09E-02
R11	[epy]Br-SIM-R1	99.48				0.52	5.03E-02
R12	[epy]Br-SIM-R2	98.70				1.30	4.15E-02
R13	[epy]Br-SIM-R3	97.44				2.56	3.22E-02
R14	[epy]Br-SIM-R4	95.01				4.99	2.26E-02
R15	[epy]Br-SIM-R5	88.38				11.62	1.24E-02
R16	[epy]Cl-DOD-R1		99.98	0.02			6.00E-02
R17	[epy]Cl-DOD-R2		99.96	0.04			4.48E-02
R18	[epy]Cl-DOD-R3		99.92	0.08			3.15E-02
R19	[epy]Cl-DOD-R4		99.85	0.15			1.97E-02
R20	[epy]Cl-DOD-R5		99.61	0.39			9.34E-03
R21	[epy]Cl-MET-R1		98.81		1.19		6.95E-02
R22	[epy]Cl-MET-R2		97.09		2.91		6.03E-02
R23	[epy]Cl-MET-R3		94.34		5.66		4.98E-02
R24	[epy]Cl-MET-R4		89.29		10.71		3.78E-02
R25	[epy]Cl-MET-R5		76.92		23.08		2.37E-02
R26	[epy]Cl-SIM-R1		99.53			0.47	6.46E-02
R27	[epy]Cl-SIM-R2		98.84			1.16	5.18E-02
R28	[epy]Cl-SIM-R3		97.70			2.30	3.91E-02
R29	[epy]Cl-SIM-R4		95.51			4.49	2.66E-02
R30	[epy]Cl-SIM-R5		89.48			10.52	1.41E-02
