# Supplementary material for: Using Delaunay triangulation and Voronoi tessellation to predict the toxicities of binary mixtures containing hormetic compound
Source: Sci Rep. 2017 Mar 13;7:43473. doi: 10.1038/srep43473 (PMC5347389; doi:10.1038/srep43473)
Supplement: Supplementary Information [file srep43473-s1.doc]

Supplementary [Information](http://www.nature.com/srep/authors/submit.html#supplementary-info) for

Using Delaunay triangulation and Voronoi tessellation to predict the toxicities of binary mixtures containing hormetic compound

Rui Qu^1^, Shu-Shen Liu^1,2,*^, Qiao-Feng Zheng^1^, Tong Li^1^

^1^ Key Laboratory of Yangtze River Water Environment, Ministry of Education, College of Environmental Science and Engineering, Tongji University, Shanghai 200092, China

^2^State Key Laboratory of Pollution Control and Resource Reuse, College of Environmental Science and Engineering, Tongji University, Shanghai 200092, China

Total pages: 18

Total tables: 2

Total figures: 6

Supplementary Material includes the following: Concentration-response models, statistics (R^2^ ad RMSE), EC_50_, and characteristic parameters (*ZEP, EC_min_* and *E_min_* for J-shape CRCs) of pesticides and ionic liquids at seven exposure times (Table S1). Some physiochemical properties, CAS number, concentration of stock, H_2_O solubility and source of five chemicals (Table S2). Concentration-response curves of DOD, MET, SIM, [epy]Br and [epy]Cl at seven exposure times (Figure S1). Concentration-response curves of 30 mixture rays in six mixture systems at seven exposure times (Figure S2). The plots of the predictive toxicities by the LMOCV based on LinIP and NeiIP vs. observation toxicities for [epy]Br-MET, [epy]Br-SIM, [epy]Cl-MET and [epy]Cl-SIM mixture systems were showed in Figure S3a and S3b. Similarly, the predictive plots by the LOOCV were showed in Figure S4a and S4b. The plots of the predictive toxicities by the LMOCV based on LinIP and NeiIP vs. observation toxicities for [emim]Br-MET, [emim]Cl-MET, [epy]Br-DOD and [epy]Cl-DOD at seven exposure times were showed in Figure S5a and S5b. Similarly, the predictive plots by LOOCV were showed in Figure S6a and S6b.

Table S1

Concentration-response models, statistics (R^2^ ad RMSE), EC_50_, and characteristic parameters (ZEP, EC_min_ and E_min_ for J-shape CRCs) of pesticides and ionic liquids at seven exposure times

| Chemical | Time  (h) | Function | R^2^ | RMSE | EC_30_  (mol/L) | EC_50_  (mol/L) | EC_70_  (mol/L) | ZEP | EC_min_ | E_min_ |
| --- | --- | --- | --- | --- | --- | --- | --- | --- | --- | --- |
| DOD | 0.25 | Weibull | 0.9930 | 0.0295 | 2.40E-06 | 4.51E-06 | 7.63E-06 |  |  |  |
|  | 2 | Weibull | 0.9860 | 0.0365 | 3.49E-06 | 6.51E-06 | 1.09E-05 |  |  |  |
|  | 4 | Weibull | 0.9815 | 0.0498 | 4.95E-06 | 7.56E-06 | 1.08E-05 |  |  |  |
|  | 6 | Weibull | 0.9899 | 0.0391 | 6.33E-06 | 8.90E-06 | 1.18E-05 |  |  |  |
|  | 8 | Weibull | 0.9898 | 0.0403 | 7.33E-06 | 1.00E-05 | 1.30E-05 |  |  |  |
|  | 10 | Weibull | 0.9956 | 0.0229 | 8.22E-06 | 1.07E-05 | 1.34E-05 |  |  |  |
|  | 12 | Weibull | 0.9953 | 0.0209 | 9.44E-06 | 1.20E-05 | 1.46E-05 |  |  |  |
| MET | 0.25 | Weibull | 0.9945 | 0.0196 | 9.82E-04 | 1.73E-03 | 2.76E-03 | - | - | - |
|  | 2 | Weibull | 0.9986 | 0.0100 | 5.74E-04 | 1.18E-03 | 2.15E-03 | - | - | - |
|  | 4 | Weibull | 0.9990 | 0.0094 | 4.93E-04 | 9.73E-04 | 1.71E-03 | - | - | - |
|  | 6 | Weibull | 0.9914 | 0.0303 | 4.78E-04 | 9.18E-04 | 1.58E-03 | - | - | - |
|  | 8 | Weibull | 0.9983 | 0.0138 | 4.22E-04 | 8.21E-04 | 1.43E-03 | - | - | - |
|  | 10 | Weibull | 0.9927 | 0.0287 | 4.57E-04 | 8.55E-04 | 1.44E-03 | - | - | - |
|  | 12 | Weibull | 0.9947 | 0.0237 | 4.93E-04 | 9.30E-04 | 1.58E-03 | - | - | - |
| SIM | 0.25 | Weibull | 0.9927 | 0.0230 | 1.02E-04 | 2.36E-04 | 4.73E-04 | - | - | - |
|  | 2 | Weibull | 0.9933 | 0.0195 | 4.56E-05 | 1.41E-04 | 3.63E-04 | - | - | - |
|  | 4 | Weibull | 0.9870 | 0.0303 | 1.02E-04 | 2.41E-04 | 4.90E-04 | - | - | - |
|  | 6 | Weibull | 0.9901 | 0.0274 | 1.26E-04 | 2.73E-04 | 5.19E-04 | - | - | - |
|  | 8 | Weibull | 0.9924 | 0.0249 | 1.52E-04 | 3.03E-04 | 5.38E-04 | - | - | - |
|  | 10 | Weibull | 0.9929 | 0.0241 | 1.83E-04 | 3.32E-04 | 5.45E-04 | - | - | - |
|  | 12 | Weibull | 0.9949 | 0.0203 | 2.09E-04 | 3.64E-04 | 5.78E-04 | - | - | - |
| [epy]Br | 0.25 | Logit | 0.9870 | 0.0364 | 5.00E-03 | 1.00E-02 | 1.72E-02 |  | - | - |
|  | 2 | Logit | 0.9664 | 0.0598 | 9.71E-03 | 1.61E-02 | 2.35E-02 |  | - | - |
|  | 4 | Logistic | 0.9746 | 0.0777 | 7.89E-03 | 1.07E-02 | 1.47E-02 | 4.51E-03 | 2.03E-03 | -14.13 |
|  | 6 | Logistic | 0.9892 | 0.0530 | 8.37E-03 | 1.07E-02 | 1.38E-02 | 5.38E-03 | 2.63E-03 | -15.67 |
|  | 8 | Logistic | 0.9953 | 0.0358 | 9.22E-03 | 1.16E-02 | 1.48E-02 | 6.19E-03 | 3.00E-03 | -19.62 |
|  | 10 | Logistic | 0.9976 | 0.0256 | 1.01E-02 | 1.26E-02 | 1.60E-02 | 6.91E-03 | 3.23E-03 | -22.76 |
|  | 12 | Logistic | 0.9941 | 0.0390 | 1.11E-02 | 1.39E-02 | 1.74E-02 | 7.39E-03 | 3.15E-03 | -24.59 |
| [epy]Cl | 0.25 | Logit | 0.9869 | 0.0410 | 4.19E-03 | 8.45E-03 | 1.48E-02 |  | - | - |
|  | 2 | Logit | 0.9670 | 0.0853 | 7.75E-03 | 1.41E-02 | 2.22E-02 |  | - | - |
|  | 4 | Logistic | 0.9750 | 0.0834 | 7.86E-03 | 1.01E-02 | 1.31E-02 | 4.81E-03 | 2.55E-03 | -11.33 |
|  | 6 | Logistic | 0.9900 | 0.0548 | 8.95E-03 | 1.11E-02 | 1.39E-02 | 6.05E-03 | 3.30E-03 | -14.96 |
|  | 8 | Logistic | 0.9923 | 0.0494 | 9.73E-03 | 1.20E-02 | 1.50E-02 | 6.74E-03 | 3.53E-03 | -18.35 |
|  | 10 | Logistic | 0.9931 | 0.0469 | 1.10E-02 | 1.35E-02 | 1.69E-02 | 7.72E-03 | 3.75E-03 | -22.61 |
|  | 12 | Logistic | 0.9950 | 0.0391 | 1.26E-02 | 1.55E-02 | 1.95E-02 | 8.92E-03 | 4.13E-03 | -26.18 |

R^2^: coefficient of determination.

RMSE: root mean square error.

ZEP: zero effect point (concentration).

E_min_: the maximum stimulatory effect or the minimum inhibition.

EC_min_: concentration with maximum stimulatory effect.

Table S2

Some physiochemical properties, CAS number , concentration of stock, H_2_O solubility and source of five chemicals.

| Chemicals | Abbr. | CAS RN | Molecular weight | Purity (%) | Source | Concentration  of stock (mol/L) | Concentration which calculated according  to the H_2_O solubility  (mol/L) | H_2_O solubility  (mg/L) |
| --- | --- | --- | --- | --- | --- | --- | --- | --- |
| dodine | DOD | 2439-10-3 | 287.4 | 98.5% | Dr.ehrenstorfer  (Germany) | 4.09E-05 | 2.19E-04 | 63 at 25 °C |
| metalaxyl | MET | 57837-19-1 | 279.3 | 98.7% | Dr.ehrenstorfer  (Germany) | 7.16E-03 | 3.01E-02 | 8400 at 22 °C |
| simetryn | SIM | 1014-70-6 | 213.3 | 97.5% | Dr.ehrenstorfer  (Germany) | 1.77E-03 | 2.11E-03 | 450 at 22 °C |
| 1-ethylpyridinium  Bromide | [epy]Br | 1906-79-2 | 188.1 | >98.0% | TCI (Japan) | 5.88E-02 | infinity | highly soluble  in water |
| 1-ethylpyridinium  Chloride | [epy]Cl | 2294-38-4 | 143.6 | >98.0% | TCI (Japan) | 7.76E-02 | infinity | highly soluble  in water |

|  |  |  |  |  |
| --- | --- | --- | --- | --- |
|  |  |  |  |  |
|  |  |  |  |  |
|  |  |  |  |  |
|  |  |  |  |  |
|  |  |  |  |  |
|  |  |  |  |  |

Figure S1 Concentration-response curves of DOD, MET, SIM, [epy]Br and [epy]Cl at seven exposure times (●: experimental values; —: CRCs fitted; : 95% CIs)

|  |  |  |  |  |
| --- | --- | --- | --- | --- |
|  |  |  |  |  |
|  |  |  |  |  |
|  |  |  |  |  |
|  |  |  |  |  |
|  |  |  |  |  |
|  |  |  |  |  |

**(a)** [epy]Br-DOD system

Figure S2 Concentration-response curves of 30 mixture rays in six mixture systems at seven exposure times (●: experimental values; —: CRCs fitted; —: CRCs predicted by CA; : 95% CIs)

|  |  |  |  |  |
| --- | --- | --- | --- | --- |
|  |  |  |  |  |
|  |  |  |  |  |
|  |  |  |  |  |
|  |  |  |  |  |
|  |  |  |  |  |
|  |  |  |  |  |

**(b)** [epy]Br-MET system

Figure S2 (continued)

|  |  |  |  |  |
| --- | --- | --- | --- | --- |
|  |  |  |  |  |
|  |  |  |  |  |
|  |  |  |  |  |
|  |  |  |  |  |
|  |  |  |  |  |
|  |  |  |  |  |

**(c)** [epy]Br-SIM system

Figure S2 (continued)

|  |  |  |  |  |
| --- | --- | --- | --- | --- |
|  |  |  |  |  |
|  |  |  |  |  |
|  |  |  |  |  |
|  |  |  |  |  |
|  |  |  |  |  |
|  |  |  |  |  |

**(d)** [epy]Cl-DOD system

Figure S2 (continued)

|  |  |  |  |  |
| --- | --- | --- | --- | --- |
|  |  |  |  |  |
|  |  |  |  |  |
|  |  |  |  |  |
|  |  |  |  |  |
|  |  |  |  |  |
|  |  |  |  |  |

**(e)** [epy]Cl-MET system

Figure S2 (continued)

|  |  |  |  |  |
| --- | --- | --- | --- | --- |
|  |  |  |  |  |
|  |  |  |  |  |
|  |  |  |  |  |
|  |  |  |  |  |
|  |  |  |  |  |
|  |  |  |  |  |

**(f)** [epy]Cl-SIM system

Figure S2 (continued)

|  |  |  |  |
| --- | --- | --- | --- |
|  |  |  |  |
|  |  |  |  |
|  |  |  |  |
|  |  |  |  |
|  |  |  |  |
|  |  |  |  |

**(a)** Based on LinIP

Figure S3 The plots of the predictive toxicities by the LMOCV vs. observation toxicities for [epy]Br-MET, [epy]Br-SIM, [epy]Cl-MET and [epy]Cl-SIM mixture systems where RMSE refers to root mean square error, R^2^ to coefficient of determination, and AR to accuracy rate.

|  |  |  |  |
| --- | --- | --- | --- |
|  |  |  |  |
|  |  |  |  |
|  |  |  |  |
|  |  |  |  |
|  |  |  |  |
|  |  |  |  |

**(b)** Based on NeiIP

Figure S3 (continued).

|  |  |  |  |
| --- | --- | --- | --- |
|  |  |  |  |
|  |  |  |  |
|  |  |  |  |
|  |  |  |  |
|  |  |  |  |
|  |  |  |  |

**(a)** Based on LinIP

Figure S4 The plots of the predictive toxicities by the LOOCV vs. observation toxicities for [epy]Br-MET, [epy]Br-SIM, [epy]Cl-MET and [epy]Cl-SIM mixture systems where RMSE refers to root mean square error, R^2^ to coefficient of determination, and AR to accuracy rate. The different colors indicate different mixture rays (■: R1, ●: R2, ▲: R3, ▼: R4, ◀: R5).

|  |  |  |  |
| --- | --- | --- | --- |
|  |  |  |  |
|  |  |  |  |
|  |  |  |  |
|  |  |  |  |
|  |  |  |  |
|  |  |  |  |

**(b)** Based on NeiIP

Figure S4 (continued).

|  |  |  |  |
| --- | --- | --- | --- |
|  |  |  |  |
|  |  |  |  |
|  |  |  |  |
|  |  |  |  |
|  |  |  |  |
|  |  |  |  |

**(a)** Base on LinIP

Figure S5 The plots of the predictive toxicities by the LMOCV vs. observation toxicities for [emim]Br-MET, [epy]Cl-MET, [epy]Br-DOD and [epy]Cl-DOD mixture systems where RMSE refers to root mean square error, R^2^ to coefficient of determination, and AR to accuracy rate.

|  |  |  |  |
| --- | --- | --- | --- |
|  |  |  |  |
|  |  |  |  |
|  |  |  |  |
|  |  |  |  |
|  |  |  |  |
|  |  |  |  |

**(b)** Based on NeiIP

Figure S5 (continued).

|  |  |  |  |
| --- | --- | --- | --- |
|  |  |  |  |
|  |  |  |  |
|  |  |  |  |
|  |  |  |  |
|  |  |  |  |
|  |  |  |  |

**(a)** Based on LinIP

Figure S6 The plots of the predictive toxicities by the LOOCV vs. observation toxicities for [emim]Br-MET, [epy]Cl-MET, [epy]Br-DOD and [epy]Cl-DOD mixture systems where RMSE refers to root mean square error, R^2^ to coefficient of determination, and AR to accuracy rate. The different colors indicate different mixture rays (■: R1, ●: R2, ▲: R3, ▼: R4, ◀: R5).

|  |  |  |  |
| --- | --- | --- | --- |
|  |  |  |  |
|  |  |  |  |
|  |  |  |  |
|  |  |  |  |
|  |  |  |  |
|  |  |  |  |

**(b)** Based on NeiIP

Figure S6 (continued).
